# D701N mutation in the PB2 protein contributes to the pathogenicity of H5N1 avian influenza viruses but not transmissibility in guinea pigs

**DOI:** 10.3389/fmicb.2014.00642

**Published:** 2014-11-25

**Authors:** Peirong Jiao, Liangmeng Wei, Yafen Song, Jin Cui, Hui Song, Lan Cao, Runyu Yuan, Kaijian Luo, Ming Liao

**Affiliations:** ^1^College of Veterinary Medicine, South China Agricultural UniversityGuangzhou, China; ^2^College of Animal Science and Veterinary Medicine, Shandong Agricultural UniversityShandong, China

**Keywords:** H5N1 HPAIV, pathogenicity, transmissibility, mice, guinea pigs

## Abstract

H5N1 highly pathogenic avian influenza virus (HPAIV) of clade 2.3.2 has been circulating in waterfowl in Southern China since 2003. Our previous studies showed that certain H5N1 HPAIV isolates within clade 2.3.2 from Southern China had high pathogenicity in different birds. Guinea pigs have been successfully used as models to evaluate the transmissibility of AIVs and other species of influenza viruses in mammalian hosts. However, few studies have reported pathogenicity and transmissibility of H5N1 HPAIVs of this clade in guinea pigs. In this study, we selected an H5N1 HPAIV isolate, A/duck/Guangdong/357/2008, to investigate the pathogenicity and transmissibility of the virus in guinea pigs. The virus had high pathogenicity in mice; additionally, it only replicated in some tissues of the guinea pigs without production of clinical signs, but was transmissible among guinea pigs. Interestingly, virus isolates from co-caged guinea pigs had the D701N mutation in the PB2 protein. These mutant viruses showed higher pathogenicity in mice and higher replication capability in guinea pigs but did not demonstrate enhanced the transmissibility among guinea pigs. These findings indicate the transmission of the H5N1 virus between mammals could induce virus mutations, and the mutant viruses might have higher pathogenicity in mammals without higher transmissibility. Therefore, the continued evaluation of the pathogenicity and transmissibility of avian influenza virus (AIVs) in mammals is critical to the understanding of the evolutionary characteristics of AIVs and the emergence of potential pandemic strains.

## INTRODUCTION

Avian influenza viruses (AIVs) are enveloped RNA viruses with an eight-segmented, single-stranded, negative-sense genome, and belong to the family *Orthomyxoviridae* (Webster et al., 1992). They are subtyped according to the characterization of hemagglutinin (HA) and neuraminidase (NA) glycoproteins, which are located on the outer surface of the virus envelope (Murti and Webster, 1986). At present, sixteen HA and nine NA subtypes have been recognized; most have been found in aquatic birds, which are the natural hosts of AIVs (Webster et al., 1992; Fouchier et al., 2005; Alexander, 2007). To date, the literature reports that only viruses of the H5 and H7 subtypes are highly pathogenic (HP) in susceptible species (Hampson and Mackenzie, 2006; Alexander, 2007).

H5N1 highly pathogenic avian influenza virus (HPAIV) isolates derived from the goose/Guangdong/1/96 (Gs/GD) lineage have been found in over sixty countries in Europe, Asia, and Africa (Li et al., 2004). They not only cause high mortality in birds and thus serious damage to the poultry industry; they also occasionally infect humans and are feared to have be the potential source of a new pandemic flu (Chen et al., 2004; Guan et al., 2004; Li et al., 2004; Peiris et al., 2007; Wang et al., 2008). H5N1 viruses are grouped into ten clades (i.e., 0–9) and many subclades based on the evolution of the HA gene. Viruses of clade 2.3.2 have been circulating widely in China since 2008 and could cause a new wave of cross-continental spread of the disease from Asia to Europe (Smith et al., 2009; Jiang et al., 2010; Li et al., 2010; Sun et al., 2011).

The most commonly used mammalian model in the study of the pathogenesis of AIVs and the evaluation of vaccines and antiviral drugs is the mouse (Gubareva et al., 1998). One clear advantage of a mouse model for AIVs infection is the wide availability of reagents for immunologic studies with mice. However, mice are not suitable for the study of the viral transmission of AIVs because infected mice fail to transmit the virus to other mice even when housed within the same cages (Lowen et al., 2006).

Unlike mice, ferrets are suitable for both transmission and pathogenesis studies, but the higher cost and large amount of space required for housing this species severely limit the number of animals that can be included in individual studies (Lowen and Palese, 2007). In an answer to this problem, the guinea pig has recently emerged as an alternate model that can be used to study the transmission of influenza viruses (Lowen et al., 2006). The guinea pig offers several advantages as a mammalian animal model for studying influenza disease, including their high susceptibility to infection with human influenza A viruses. In addition, their lungs contain bronchus-associated lymphoid tissue similar to that of humans, and the airway innervation of the guinea pig is very similar to that of humans as well (Azoulay-Dupuis et al., 1984; Wang et al., 2005). Guinea pigs have been successfully used as models to evaluate the transmissibility of AIVs and other influenza viruses in mammalian hosts (Lowen et al., 2006; Bouvier et al., 2008; Lowen et al., 2009; Mubareka et al., 2009; Steel et al., 2009; Van Hoeven et al., 2009).

Our previous studies demonstrated that a large number of H5N1 HPAIVs within clade 2.3.2 from Southern China had high pathogenicity in different birds (Sun et al., 2011; Yuan et al., 2014). Some studies have investigated the pathogenicity and transmissibility of H5N1 HPAIVs of this clade in mammals (Hu et al., 2013; Xu et al., 2013). To better understand the pathogenicity of these viruses in mice and guinea pigs and the transmissibility in guinea pigs, we selected a clade 2.3.2 virus isolated in 2008 from ducks to examine its lethality, replication and transmission in these animals.

## MATERIALS AND METHODS

### VIRUS

The A/duck/Guangdong/357/2008 (DK357) virus used in this study was isolated from ducks in the Guangdong Province of China in 2008 and identified as H5N1 AIV by means of HA inhibition and NA inhibition tests. The virus was purified and propagated in the allantoic cavity of 10-day-old, specific-pathogen-free (SPF), embryonated hens’ eggs. The allantoic fluid from multiple eggs was pooled, clarified by centrifugation, and frozen in aliquots at -70°C. The 50% egg infectious dose (EID_50_) was calculated according to the method published by Reed and Muench (1938) using the serial titration of eggs. All experiments were carried out in Animal Biosafety Level 3 (ABSL-3) facilities.

### ANIMALS

Six-week-old female BALB/c mice and female Hartley strain guinea pigs weighing 300–350 g were purchased from the Laboratory Animal Center of South China in Guangzhou, China. The mice and guinea pigs were confirmed as serologically negative for the AIVs used in these studies. The animals were housed in ABSL-3 facilities.

### MOUSE EXPERIMENTS

To determine morbidity and mortality, groups of eight 6-week-old female BALB/c mice were lightly anesthetized with CO_2_ and inoculated intranasally with 10^6^ EID_50_ of virus in a volume of 0.05 ml. Additionally, five mice were inoculated with 0.05 ml of phosphate buffered saline (PBS) and served as the sham control group. Three mice in each group were euthanized at 3 days post-inoculation (DPI), and the lungs, kidneys, spleens, and brains were collected for virus titration in eggs as described previously (Chen et al., 2004; Jiao et al., 2007). The remaining mice were monitored daily for weight loss and mortality to 14 DPI. Mice that lost more than 25% of their original weight were euthanized for humane reasons. All animal experiments were conducted under the guidance of CDC’s Institutional Animal Care and Use Committee and in an Association for Assessment and Accreditation of Laboratory Animal Care International accredited facility. Our animal experiments in this study had been approved by Guangdong Province Animal Disease Control Center and were carried out in ABSL-3 facilities.

### GUINEA PIG EXPERIMENTS

In this study, intramuscular injections of ketamine (20 mg/kg) and xylazine (1 mg/kg) were used to anesthetize guinea pigs. To determine morbidity and mortality, groups of eight animals were anesthetized, and a 0.3 ml volume of inoculum containing 10^6^ EID_50_ of virus was instilled into the nostrils (0.15 ml each side) of each guinea pig in the experimental groups. Five guinea pigs were inoculated with 0.3 ml PBS and acted as the sham control group. Three guinea pigs in each group were euthanized at 3 DPI, and the nasal turbinates, tracheas, lungs, brains, kidneys, spleens, livers, and colons were collected for virus titration in eggs following the previously described method (Chen et al., 2004). The remaining animals were monitored daily for weight loss and mortality up to 14 DPI. All animal experiments were conducted under the guidance of CDC’s Institutional Animal Care and Use Committee and in an Association for Assessment and Accreditation of Laboratory Animal Care International accredited facility. Our animal experiments in this study had been approved by Guangdong Province Animal Disease Control Center and were carried out in ABSL-3 facilities.

### CONTACT TRANSMISSION EXPERIMENTS BETWEEN GUINEA PIGS

To assess the transmissibility of the virus among guinea pigs, three additional animals were inoculated intranasally with 10^6^ EID_50_ of the test virus and housed in a cage. Five naive animals were introduced into the same cage 24 h later. Three naive animals were euthanized at 5 days post contact (DPC), and their organs were collected for virus titration in eggs following the previously described method (Chen et al., 2004). The remaining animals were monitored daily for weight loss and mortality up to 14 DPC. All animal experiments were conducted under the guidance of CDC’s Institutional Animal Care and Use Committee and in an Association for Assessment and Accreditation of Laboratory Animal Care International accredited facility. Our animal experiments in this study had been approved by Guangdong Province Animal Disease Control Center and were carried out in ABSL-3 facilities.

### MOLECULAR CHANGE ANALYSIS

The viral RNA was extracted from the allantoic fluid supernatant using the RNeasy Mini Kit (Promega, Madison, WI, USA), and the manufacturer’s instructions were followed. Reverse transcription polymerase chain reaction (RT-PCR) was conducted using the Superscript III (Invitrogen, Carlsbad, CA, USA) and Uni12 (5′-AGCAAAAGCAGG-3′) primer. Eight genes were amplified using universal primers (Hoffmann et al., 2001), and the PCR products were purified using the mini PCR Purification Kit (Promega). Sequencing was performed by Shanghai Invitrogen Biotechnology Co., Ltd. The sequencing data were compiled with the Seqman program of Lasergene 7 (DNASTAR, Inc.). Amino acid sequence similarities were identified with the Lasergene 7 Megalign program (DNASTAR).

## RESULTS

### VIRUS PATHOGENICITY AND ORGAN TROPISM IN THE MOUSE MODEL

**FIGURE 1:**
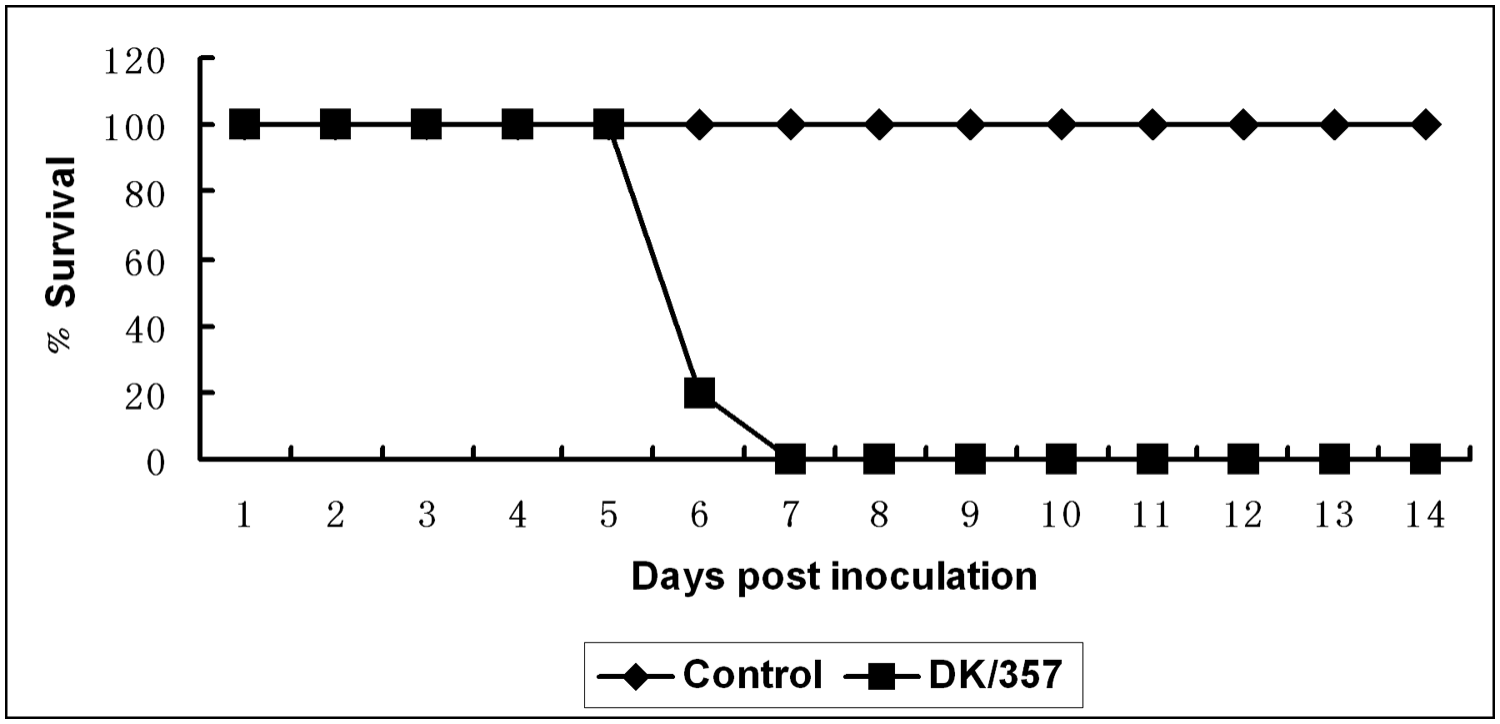
**Lethality of the DK/357 virus to mice.** Survival curves of the mice inoculated with DK/357 virus at a dose of 10^6^ EID_50_.

In the mouse experiment, clinical signs of illness were observed in the experimental group as early as 3 DPI, and all mice exposed to the virus were dead by 7 DPI (**Figure 1**). For the three mice euthanized on 3 DPI, the mean virus titer was 5.8, 3.8, 3.3, and 3.9 log_10_EID_50_ in the lung, spleen, kidney, and brain, respectively (**Table 1**). Infection with the DK/357 virus caused 23.1% loss of body weight by 7 DPI, while the sham control group inoculated with PBS increased in body weight by an average of 11.4% by 14 DPI. These findings illustrated that DK/357 virus had high pathogenicity in mice.

**Table 1:** Replication of H5N1 avian influenza viruses in mice and guinea pigs.

Virus strains	Virustiters in mice at 3 DPI (log_10_EID_50_/g)				Virus titers in guinea pigs at 3 DPI (log_10_EID_50_/g)					
	Lung	Spleen	Kidney	Brain	Nasal turbinate	Trachea	Lung	Brain	Kidney	Spleens	Livers	Colon
DK/357	5.8 ± 1.1^a^	3.8 ± 0.9	3.3 ± 0.8	3.9 ± 1.1	5.8 ± 1.5	2.6 ± 0.8	5.6 ± 1.4	–^b^	–	–	–	–
DK/357-T	7 ± 1.4	3.9 ± 1.1	3.6 ± 0.9	4.3 ± 0.9	6.8 ± 1.2	2.8 ± 0.8	6 ± 1.6	1.9 ± 0.7	–	–	–	–
DK/357-L	6.6 ± 1.2	3.4 ± 0.9	2.9 ± 1.2	3.9 ± 1.2	6 ± 1.3	2.2 ± 0.6	6.3 ± 1.3	–	–	–	–	–

*A value of 1 was assigned if the virus was detected from the undiluted sample of mice in three embryonated hen eggs and 1.5 was assigned if the virus was detected from the undiluted sample of guinea pigs. ^a^Virus titers are expressed as means ± SD in log_10_EID_50_/g of tissue. ^b^No detected.*

### VIRUS PATHOGENICITY AND ORGAN TROPISM IN THE GUINEA PIG MODEL

In the mice studies, DK/357 was HP, i.e., causing 100% morbidity and mortality. In contrast, in guinea pig model, infection with the virus did not cause morbidity. What’s more, infection with the DK/357 virus only caused slight weight loss in guinea pigs during the initial DPI. In the evaluation of the organ tropism of the virus in the guinea pigs, the virus was found in the nasal turbinates, tracheas, and lungs with mean titers of 5.8, 2.6, and 5.6 log_10_EID_50_, respectively (**Table 1**). No virus was isolated from any of the other collected guinea pig tissues. These results indicated that DK/357 virus only replicated in some tissues of the guinea pigs without production of clinical signs.

### TRANSMISSION OF THE VIRUS BETWEEN CO-CAGED GUINEA PIGS

In the investigation of virus transmission among co-caged guinea pigs, the virus was isolated only from the nasal turbinates of two guinea pigs with titers of 2.75 and 2 log_10_EID_50_, respectively, at 5 DPC. At 9 DPC, one co-caged guinea pig showed slight clinical symptoms. This guinea pig was euthanized, and samples were collected for virus titration. The virus was isolated from the trachea (named DK/357-T) and the lung (named DK/357-L) with titers of 1.75 and 3 log_10_EID_50_, respectively.

### MOLECULAR CHANGE ANALYSIS BETWEEN ORIGINAL VIRUS ISOLATES AND VIRUSES ISOLATED FROM THE TRACHEA AND LUNG OF THE CO-CAGED GUINEA PIG

Viral RNA was extracted from the trachea and lung of the co-caged guinea pig, and RT-PCR was conducted. The PCR products of eight genes from the viral RNA extracted from the trachea and lung of the co-caged guinea pig were sequenced and were compared to the sequence of the original virus isolate in order to identify any molecular differences between the two. When comparing DK/357 to the DK/357-T, three amino acids substitutions were found, i.e., V96I and D701N in the polymerase PB2 protein and N417S in the nucleoprotein (NP) protein (**Table 2**). In total, the following five amino acid changes between DK/357 and DK/357-L were identified: V96I, F135I, S688F, and D701N in the polymerase PB2 protein and N417S in the NP protein (**Table 2**).

**Table 2:** The amino acids substitution among DK/357, DK/357-T, and DK/357-L.

	Amino acid substitution				
Viruses	NP	PB2			
	417	96	135	688	701
DK/357	N	V	F	S	D
DK/357-T	S	I	F	S	N
DK/357-L	S	I	Y	F	N

*Amino acids sites were located by taking A/Gs/GD/1/96 as reference.*

### PATHOGENICITY AND ORGAN TROPISM OF DK/357-T AND DK/357-L IN THE MOUSE AND GUINEA PIG MODELS

**FIGURE 2:**
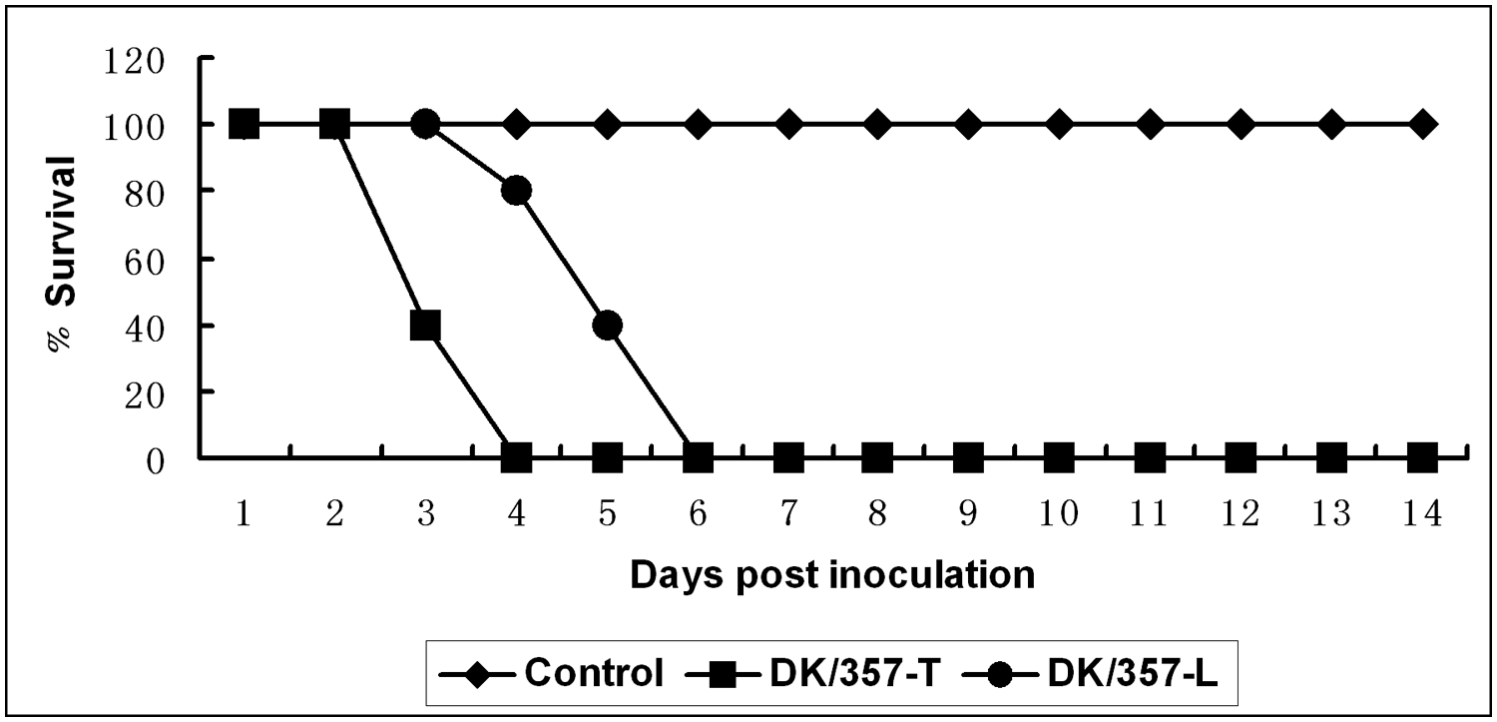
**Lethality of the DK/357-T and DK/357-L viruses to mice.** Survival curves of the mice inoculated with DK/357-T and DK/357-L viruses at a dose of 10^6^ EID_50_.

All mice exposed to the DK/357-T and DK/357-L were dead by 4 and 6 DPI, respectively (**Figure 2**). The DK/357-T virus replicated systemically to the mean titers of 7, 3.9, 3.6, and 4.3 log_10_EID_50_ in the lung, spleen, kidney, and brain, respectively (**Table 1**). The virus titers in the detected tissues were all higher than that of the original DK/357 virus. The DK/357-L virus was replicated to the mean titers of 6.6, 3.4, 2.9, and 3.9 logEID_50_ in the lung, spleen, kidney, and brain, respectively (**Table 1**). The mean virus titers were higher than that in the lung and brain after infection with the original DK/357 virus but lower in the spleen and brain than that after infection with the original DK/357 virus.

To investigate the morbidity and mortality of DK/357-T and DK/357-L in guinea pigs, the previously described intranasal inoculation procedures were performed using DK/357-T and DK/357-L for the virus inoculum with groups of eight guinea pigs for each. As observed in the original DK/357 virus, neither of the viruses caused morbidity in guinea pigs. In the organs of the three sacrificed animals from each group on 3 DPI, the DK/357-T virus had replicated in the nasal turbinates, tracheas, and lungs with mean titers of 6.8, 2.8, and 6 log_10_EID_50_, respectively (**Table 1**). The virus was even found in the brain of one guinea pig with a titer of 2.7 log_10_EID_50_. After infection with the DK/357-L virus, the virus was also found in the nasal turbinates, tracheas, and lungs of the sacrificed animals with mean titers of 6, 2.2, and 6.3 log_10_EID_50_, respectively (**Table 1**). Overall, the replication ability of the two viruses was higher than the original virus isolate; however, the mean titer value in the trachea of the guinea pigs infected with DK/357-L was lower than that of the guinea pigs infected with the original DK/357 isolate.

### TRANSMISSION ABILITY OF THE DK/357-T AND DK/357-L BETWEEN CO-CAGED GUINEA PIGS

In order to investigate the transmission ability of DK/357-T and DK/357-L in guinea pigs, the previously described guinea pig inoculation and co-cage procedures were repeated using DK/357-T and DK/357-L. At 5 DPC, the virus was only detected in the nasal turbinates of two of the guinea pigs co-caged with the guinea pigs inoculated with the DK/357-T with a mean titer 2.1 logEID_50._ In comparison, the virus was only detected in the nasal turbinates of one guinea pig co-caged with the guinea pigs inoculated with the DK/357-L with a mean titer 1.8 log_10_EID_50_.

## DISCUSSION

From 2003 through 2 October, 2014, 668 laboratory-confirmed human cases of H5N1 AIV infection have been officially reported to WHO from 16 countries. Of these cases, 393 have died (http://www.who.int/). Most patients who had confirmed infections of H5N1 HPAIV died of respiratory failure complicated by acute respiratory distress syndrome; in most instances, the viral infection resulted from direct exposure to poultry or poultry products infected with H5N1 HPAIV (Beigel et al., 2005). However, some very limited human-to-human transmission has been detected (Ungchusak et al., 2005). The H5N1 viruses of clade 2.3.2 increased in prevalence in poultry and wild birds and became the dominant clade, especially in the mainland of China and Hong Kong during 2008–2009 (Smith et al., 2009; Li et al., 2010). We also isolated some H5N1 HPAIV viruses of clade 2.3.2 in 2008 and 2009. This study was undertaken to determine the pathogenicity of the virus of this period to mice and guinea pigs and the transmissibility between guinea pigs.

Mice have previously been used as a mammalian animal model when evaluating the pathogenicity of the influenza virus (Gubareva et al., 1998). In this study, mice were highly susceptible to infection with the DK/357 virus; in fact, all mice exposed to the virus died by 7 DPI, i.e., 100% morbidity and mortality. The virus was isolated from all harvested tissues at relatively high virus titers. In contrast, when guinea pigs were inoculated with the DK/357 virus, none of guinea pigs died, but the virus was able to replicate in some tissues of the guinea pigs (**Table 1**). Our findings indicated that the pathogenicity of the DK/357 virus in mice and guinea pigs was different.

Like humans and ferrets, guinea pigs can be productively infected by human (i.e., unadapted) influenza virus isolates, develop an upper respiratory tract infection, and transmit the virus to other guinea pigs in both contact and non-contact situations (Lowen et al., 2006). In addition, guinea pigs have been successfully used as models to evaluate the transmissibility of AIVs and other influenza viruses in mammalian hosts (Lowen et al., 2006; Bouvier et al., 2008; Lowen et al., 2009; Mubareka et al., 2009; Steel et al., 2009; Van Hoeven et al., 2009). In this study, the DK/357 isolate could replicate in some tissues of the guinea pigs but did not cause morbidity. The transmission experiment results showed the virus could be transmitted among guinea pigs. Interestingly, one co-caged guinea pig showed slight clinical symptoms at 9 DPC. This guinea pig was euthanized, and the virus was isolated from the trachea (i.e., designated DK/357-T) and lung (i.e., designated DK/357-T). Sequenced results showed N417S mutation in the NP protein and V96I, D701N mutation in the PB2 protein in these two viruses (**Table 2**). Previous researches have reported that V96I in the PB2 protein and N417S in the NP protein also existed in low pathogenic human and/or AIV, and these mutations did not enhance their pathogencity to mice (Nolting, 2008; Qi et al., 2012; Aguirre et al., 2014). The D701N mutation in the PB2 protein is known to affect the replicative efficiency of H5N1 influenza A viruses in mice and transmissibility in guinea pigs (Li et al., 2005; Gao et al., 2009; Steel et al., 2009; Gabriel et al., 2013; Czudai-Matwich et al., 2014). These previous reports prompted us to evaluate the pathogenicity of these two viruses in mice and guinea pigs and their transmissibility among guinea pigs.

The DK/357-T and DK/357-L isolates manifested an increased pathogenicity in mice; indeed, they caused more severe weight loss and demonstrated stronger replication capability than DK/357. Although the replication capability of DK/357-T and DK/357-L was a little higher than that of DK/357 in guinea pigs (**Table 1**), they were not lethal to guinea pigs. In addition, when guinea pigs were inoculated with DK/357-T, the virus was found in the brain of one guinea pig. Czudai-Matwich et al. (2014) reported that mutation D701N led to an increase in polymerase activity and replication efficiency in mammalian cells and in mouse pathogenicity, and this increase was significantly enhanced when mutation D701N was combined with mutation S714R. Steel et al. (2009) found that when PB2 627 holds a glutamic acid residue, the D701N mutation not only improves viral growth in mammalian cells but enhances transmission of both human influenza viruses and AIVs among guinea pigs. Multi-genes of H5N1 AIV would affect virus’s ability to transmission among the mammals. Gao et al. (2009) reported the 701N in the PB2 protein was a prerequisite for A/duck/Guangxi/35/01 transmission in guinea pigs. However, an amino acid change in the HA protein (T160A), resulting in the loss of glycosylation at 158–160, was responsible for HA binding to sialylated glycans and was critical for H5N1 virus transmission in guinea pigs (Gao et al., 2009). In our study, the D701N mutation in PB2 protein did not enhance the virus’s transmission ability among guinea pigs which suggest other genes may affect virus’s ability to transmission among the guinea pigs.

In summary, our findings illustrated that the H5N1 duck-origin influenza virus that belongs to the 2.3.2 clade demonstrated high pathogenicity in mice. This virus only replicated in some tissues of the guinea pigs without production of clinical signs but could be transmitted among guinea pigs. Interestingly, the viruses isolated from the co-caged guinea pigs had a D701N mutation in the PB2 protein. These mutant viruses showed higher pathogenicity in mice and higher replication capability in guinea pigs; however, these viruses did not show enhanced transmissibility among guinea pigs. Therefore, the continued evaluation of the pathogenicity and transmissibility of AIVs in mammals is critical to the understanding of the evolutionary characteristics of AIVs and the emergence of potential pandemic strains.

## Conflict of Interest Statement

The authors declare that the research was conducted in the absence of any commercial or financial relationships that could be construed as a potential conflict of interest.
